# Psychosocial stressors, accelerated biological aging, and multiple morbidities: Evidence from an age-diverse sample

**DOI:** 10.1371/journal.pone.0343987

**Published:** 2026-03-06

**Authors:** Gabriele Ciciurkaite, Byungkyu Lee, Siyun Peng, Maleah Fekete, Colter Mitchell, Brea L. Perry

**Affiliations:** 1 School of Social Sciences, Utah State University, Logan, Utah, United States of America; 2 Department of Sociology, New York University, New York City, New York, United States of America; 3 School of Aging Studies, University of South Florida, Miami, Florida, United States of America; 4 Department of Sociology and the Irsay Institute for Sociomedical Sciences Research, Indiana University, Bloomington, Indiana,; 5 Institute for Social Research, University of Michigan, Ann Arbor, Michigan, United States of America; University of Newcastle, UNITED KINGDOM OF GREAT BRITAIN AND NORTHERN IRELAND

## Abstract

Exposure to psychosocial stress is a well-established risk factor for poor health and premature mortality, yet most research has focused narrowly on single sources of stress without simultaneously modeling multiple stress exposures occurring across the life span. Using data from a state-representative sample of 2,267 adults ages 18–103, we examined associations between four psychosocial stressors – adverse childhood experiences (ACEs), stressful life events, chronic financial strains, and everyday discrimination – and DNA methylation-based biological aging clocks (GrimAge2 and DunedinPACE) alongside six indicators of physical and mental health outcomes. All stressors were associated with accelerated epigenetic aging and poorer health when examined individually. However, when considered simultaneously, financial strains and everyday discrimination emerged as more consistent predictors across all outcomes, relative to childhood adversity and stressful events in adulthood. Overall, stressor effects were more pronounced for mental health compared to physical health or biological aging. These findings highlight the importance of considering multiple sources of stress on varying indicators of aging, disease, and distress to fully account for the health significance of stress exposure.

## Introduction

Exposure to psychosocial stress – broadly defined as challenging circumstances triggering behavioral and physiological readjustment – elevates risk for poor physical and mental health, and premature mortality [1–7]. These associations often reflect the biological embedding of day-to-day social experiences, wherein emotional and physical threats emerge from structurally patterned exposures rooted in key institutional domains including intimate relationships, labor markets, and the political economy. For example, about 58% of American adults report having experienced at least one form of adversity in their childhood [8], around 40% of US adults aged 33–44 years have experienced at least one familial loss [9], about 30% of the general population experience at least one type of major discrimination in their lifetime [10], and about 12% of American households experience food insecurity in a given year [11]. Research suggests that the cumulative load of such experiences may lead to dysregulation of stress response systems [12,13].

The allostatic load framework helps explain how these diverse stressors become biologically embedded [12]. Psychosocial stressors trigger regulatory physiological responses across multiple bodily systems. This process begins with the release of cortisol and catecholamines (epinephrine and norepinephrine), which in turn initiate adaptive responses in the cardiovascular, metabolic, and immune systems. After the threat ceases, the physiological response is shut off. Such activation is protective in the short-term, however, prolonged or repeated exposure to stress overwhelms these systems and erodes their capacity to regulate effectively, leading to greater allostatic load. Allostatic load, thus, is the physiological toll that the body carries due to repeated exposure to one or more stressors, which accumulates over the life course. This physiological toll or “wear and tear” does not represent poor health, but may increase risk for early disease onset and undermine healthy aging [12,14]. Consistent with this framework, individuals facing multiple and/or ongoing stressors are likely to experience greater physiological burden.

Recent advances in epigenetics have enabled direct measurement of said “wear and tear” on the molecular level [15]. Specifically, DNA methylation accumulates across the genome with age and in response to social and environmental exposures, creating an epigenetic signature that is unique to each individual. Epigenetic clocks are generated using machine learning algorithms that identify patterns in DNA methylation data at specific sites on the genome to predict biological age. These can be compared against chronological age to reflect accelerated aging processes [16,17]. Growing evidence indicates that stressor exposure across the life span accelerates epigenetic aging, affecting gene expression and increasing risk for a number of aging-related disease outcomes and all-cause mortality [17]. However, this empirical evidence comes from studies generally focused on a single type of stressor occurring at one point in the life span and a single health outcome. This approach is limited because: 1) stressors beget stressors, and the true effect of any single source can only be identified by accounting for the full range of exposures [18–23]; 2) no single type of stressor is sufficient to capture the total impact of stressful experiences on biological aging [24]; and 3) it leaves critical questions about whether the effects of stress exposure on biological aging extend across other objectively and subjectively measured indicators of health status or exhibit outcome-specific patterns of association [25].

In the psychosocial approach, researchers have argued for modeling different sources of stress in the same analytical framework [18,26]. Typically, sources of stress have been categorized based on timescale and life stage of exposure [18,26,27]. Adverse childhood experiences (ACEs), including abuse, neglect, and household dysfunction, occurring during critical developmental periods, have received much of the scholarly focus, with evidence documenting the broadest and most durable impact on many forms of morbidity, as well as accelerated biological aging [28–32]. Stressful life events and traumas experienced in adulthood, such as divorce, job loss, or death of loved ones, represent acute disruptions and have also been linked to biological age acceleration and onset and progression of diseases [4,33–36]. Likewise, prolonged exposure to everyday psychosocial stressors in adulthood, such as financial or relationship strains and interpersonal discrimination, leads to a state of constant stimulation and hypervigilance, overwhelming regulatory systems and increasing risk for physical and mental health disorders, as well as shortened health span and life span [36–44].

Despite robust evidence that psychosocial stress contributes to poorer health, critical gaps remain. First, because most studies examine a single type of stressor on a particular health outcome in isolation, their relative importance on broad indicators of health remains less well understood. It is a critical knowledge gap given that different sources of stress represent distinct dimensions of social experiences with varying effects on psychological distress [24,45], inflammatory gene expression [21], immune aging [36], and epigenetic aging [42,44,46]. Second, existing studies rarely focus on the timing of stressor exposure within the life course; while childhood and adult adversities have mostly been studied, few studies consider them together. Although it is well established that early life adversities have far-reaching health implications [28,47], it remains unclear whether the effects of early life adversities persist after accounting for adversities experienced in adulthood [21,23]. This is a critical oversight because the observed associations might reflect the impact of other stressors that are collinear, and the true cause of the outcome may only be elucidated when accounting for exposures that childhood adversity may beget. Finally, research on epigenetic mechanisms of stress-related morbidity has been limited by the lack of representative samples spanning the full adult age range, as most studies rely on biomarker data from mid- and late adulthood populations, raising questions about the replicability of findings [43,48–52] (although see [42,44,53] for notable exceptions).

To address these gaps, we leverage a large, age-heterogeneous (ages 18–103), and socioeconomically diverse sample of Indiana residents to comprehensively assess the effects of psychosocial stress experienced across different moments in the life course and eight health indicators: accelerated biological aging (GrimAge2 and DunedinPACE), physical health (self-rated health, multimorbidity, and pain interference), and self-reported mental health, as well as depression and anxiety severity as measured by the Computerized Adaptive Test – Mental Health (CAT – MH) [54,55]. These health indicators have all been previously considered in separate strands of stress research but never examined simultaneously [4,56]. Considering that past research has documented independent variance in health outcomes associated with various stressors, we differentiate between four sources of psychosocial stress based on life stage of exposure and timescale – adverse childhood experiences (ACEs), stressful life events, chronic financial strain, and everyday discrimination [21,23,24,33,36,44,57]. We analyze discrimination as a distinct source of stress following established best practices [36,37]. We examine how socially patterned stress exposures become biologically embedded by considering stressors experienced in multiple domains and examining their impact on a range of health outcomes. This approach provides a more comprehensive understanding of interrelated social, psychological, and biological processes.

## Methods

We utilize data from the Person to Person Health Interview Study (P2P), which is an omnibus health survey that uses a stratified probability sample of households across the state of Indiana. Sampling, recruitment, and survey methodology for the baseline interview were designed to parallel the nationally representative General Social Survey. Participants were noninstitutionalized, cognitively capable adults aged 18 years and older. Interview and anthropometric data were collected face-to-face by rigorously trained interviewers employed by the Indiana University Center for Survey Research. Of the survey participants (N = 2,435), 91% agreed to provide their saliva for epigenetic analysis, following the prescribed protocols of the DNA Genotek Oragene guidelines. DNA was extracted and checked for quality within 6 weeks of collection by the Indiana University Genetics Biobank using Chemagic 360 magnetic bead-based high throughput extraction. All data collection took place between October 2018 and July 2021. Written informed consent was obtained for all data collection, and the privacy rights of human subjects were observed. All procedures were performed in compliance with relevant institutional guidelines and have been approved by Institutional Review Board at Utah State University (Protocol #14680). Interview data were collected for 2,685 respondents (response rate about 30%), and biomarker data were available for 2,347 of them after epigenetic processing and quality control. An additional 80 cases were dropped due to missing data on other predictors and outcome variables, yielding the final analytical sample of N = 2,267. For the purposes of the analyses, de-identified data were accessed on 11/01/2024.

## Measures

### Epigenetic aging

Epigenetic age was measured using DNAm-GrimAge2 and DunedinPACE (PACE) clocks. The development of GrimAge2 followed a two-stage approach with Framingham Heart Study data (N = 2,544 individuals aged 40–92 years, split into training [N = 1,833] and test [N = 711] datasets). Stage 1 involved creating DNA methylation surrogates for high-sensitivity C-reactive protein and hemoglobin A1C using elastic net regression with 10-fold cross-validation. Stage 2 employed an elastic net Cox proportional hazards model to predict all-cause mortality using chronological age, sex, and ten DNA methylation-based biomarkers (the two new protein surrogates plus eight from the original GrimAge including smoking pack-years and various plasma proteins). The mortality risk estimate was linearly transformed into units of years to create an interpretable biological age measure. The model was validated across 13,399 blood samples from nine independent cohorts representing multiple racial/ethnic groups, demonstrating superior performance compared to the original GrimAge in predicting mortality and age-related health conditions [58]. In this study, we use the age-adjusted version (i.e., age regressed out), which measures accelerated (positive residuals) biological aging relative to chronological age (AgeAccelGrim2).

DunedinPACE is a measure of rate of aging. The creation of this measure also involved multiple phases. First, longitudinal changes in 19 biomarkers related to multiple organ function were measured at ages 26, 32, 38, and 45. Next, linear mixed effects models were used to estimate participants’ individual rate of change for each of the 19 biomarkers, and then these 19 rates of change for individual biomarkers were combined to create individual-level biomarkers, or pace of aging. This pace of aging was then scaled so that it could be interpreted in reference to a base rate of 1 biological year per chronological year. Finally, elastic-net-regression was used to generate an algorithm that predicts 20-year pace of aging, using a subset of DNA methylation data from participants at age 45 [59].

### Physical health outcomes

Self-rated physical health was measured by asking the respondents if they perceived their physical health as 1) Poor; 2) Fair; 3) Good; 4) Very good; or 5) Excellent. Multimorbidity (range = 0 – 9) was measured by counting the self-reported number of conditions diagnosed by a physician among arthritis, asthma, cancer, emphysema, stroke, myocardial infarction, coronary heart disease, kidney disease, obesity, and diabetes. Pain interference was measured by asking the respondents to rate the extent to which pain interferes with their general level of activity on a scale from 0 to 10.

### Mental health outcomes

Self-rated mental health was measured by asking the respondents if they perceived their mental health as 1) Poor; 2) Fair; 3) Good; 4) Very good; or 5) Excellent. Depression and anxiety severity (range  =  0 – 100) were measured using the Computerized Adaptive Test ‒ Mental Health (CAT – MH) [54,55], an efficient computerized adaptive test based on multidimensional item response theory. The CAT – MH contains validated measures for diagnostic screening and continuous measurement of targeted mental health disorders and is superior to brief assessments used in other studies.

### Psychosocial stressors

We considered four sources of social stress – adverse childhood experiences (ACEs), stressful life events, chronic financial strains, and everyday discrimination. ACEs were measured using the 10-item index, which assesses potentially traumatic events that occur in childhood (0 – 17 years), such as experiencing violence, abuse, or neglect, witnessing violence in the home, or substance abuse in the home [32] (Cronbach’s α = 0.83). Stressful life events (range = 0 – 9) included a total count of self-reported experiences of loss of a child, loss of a mother, loss of a father, loss of a sibling, divorce, widowhood, criminal arrest, probation or parole, not hired for unfair reasons, fired or denied promotion for unfair reasons, and unfairly stopped, searched, questioned, physically threatened, or abused by the police. Chronic financial strains (range = 0 – 7) included a total count of self-reported ongoing experiences of lack of money to buy food, reducing the size of meals or skipping meals, or not having enough money for shelter or clothing in the past 6 months, currently renting a home (versus owning), experiencing crowding and limited space at home, experiencing unstable housing, or being unemployed, and having missed doctor appointments due to limited finances in the past 12 months. Everyday discrimination [60] was assessed by asking respondents how often they experienced various forms of mistreatment, such as being called names or insulted, threatened or harassed, treated with less courtesy or respect compared to others, receiving poorer service in restaurants or stores, encountering people who act afraid of them, or being perceived as unintelligent, dishonest, or inferior. Additionally, respondents were asked if they were followed in stores. Responses ranged from (0) Never to [5] Every day and were summed to create a composite measure (range = 0 – 45). Cronbach’s α for this measure was 0.86.

### Control variables

Gender is a 2-category measure and includes female and male (reference). Race/ethnicity is a three-category measure, which includes White, Black, and Other (reference) categories. Educational attainment is a 4-category variable that includes less than high school (reference), high school or GED, some college, and bachelor’s degree or higher. We also controlled for age (measured as a continuous variable). To account for potential differences in epigenetic aging measures associated with technical variables, we also controlled for DNA methylation assay batch and leukocyte proportion in salivary cells. We created estimates for Leukocyte and Epithelial cell proportions with the estimateLC function from the ewastools R package, using the Middleton saliva reference panel for deconvolution. These proportions sum to one, therefore only one variable can be included in models to avoid collinearity [61]. Due to the outbreak of the COVID-19 pandemic, P2P data collection stopped on March 19, 2020, and resumed on July 17, 2020, when it was deemed safe. To account for potential differences associated with data being collected before or after the pandemic, we created a binary variable to indicate this.

### Analytic strategies

To examine the association between each of the four sources of stress exposure and eight health outcomes, we ran a series of multivariate standardized OLS regression models. In the unadjusted models, we systematically examined the effect of each stressor, adjusting for control variables only. Because we ran a total of 32 regression models, p-values were corrected for multiple testing using the Benjamini-Hochberg False Discovery Rate (FDR) procedure with Q = 0.05 in sensitivity analyses. All the results remained significant after the correction. Then, to consider the relative effect of different stressors on each health outcome, we simultaneously examined all sources of stress in eight adjusted regression models. Considering that different sources of stress are correlated, we assessed the Variance Inflation Factor (VIF) scores after running each regression to test for multicollinearity; however, no VIF score exceeded 3.5. Every regression model controlled for age, gender, race/ethnicity, and educational attainment. Models predicting epigenetic aging additionally controlled for epigenetic assay batch and leukocyte proportion in salivary cells. We applied survey weights to all regression analyses. Analyses were conducted in Stata 19.

## Results

Weighted descriptive statistics are provided in Table 1. The chronological age of study participants ranged from 18 to 103 (mean = 45.45, SD = 18.09), providing a heterogeneous age range to examine the effects of stressors on various health indicators. The mean biological age (DNAm GrimAge2) was 65.10 (SD = 15.85), and the mean pace of aging (DunedinPACE) was 1.19 years per every chronological year (SD = 0.19), reflecting a biologically older sample relative to the chronological age. An average participant reported 2.22 (SD = 2.36) adverse events in their childhood, 2.40 (SD = 1.92) stressful adulthood life events, 1.20 (SD = 1.49) chronic financial strains, and a mean everyday discrimination scale score of 8.03 (SD = 7.38). The majority of the participants (75.26%) reported excellent, very good, or good physical health, 1.08 (SD = 1.30) physical health diagnoses, and a pain interference score of 3.16 (SD = 2.84). Further, most of the participants (81.99%) reported excellent, very good, or good mental health, a mean depression severity score of 29.31 (SD = 19.14), and a mean anxiety severity score of 18.85 (SD = 18.34).

**Table 1:** Descriptive statistics of the study sample.

	Mean or %	SD	Range
*Biological Aging Measures:*			
GrimAge2^a,1^	65.10	15.85	34.33–129.25
DunedinPACE^b^	1.19	0.19	0.73–1.93
*Physical Health Measures:*			
Self-Rated Physical Health			
Excellent	10.00%		
Very good	27.16%		
Good	38.10%		
Fair	18.97%		
Poor	5.77%		
Multimorbidity	1.08	1.30	0–9
Pain interference	3.16	2.84	1–10
*Mental Health Measures:*			
Self-Rated Mental Health:			
Excellent	16.62%		
Very good	34.01%		
Good	31.36%		
Fair	13.64%		
Poor	4.38%		
Depression severity	29.31	19.14	0–100
Anxiety severity	18.85	18.34	0–100
*Stress Exposure Measures:*			
Adverse Childhood Experiences (ACEs)	2.22	2.36	0–10
Stressful life events	2.40	1.92	0–9
Chronic financial strains	1.20	1.49	0–7
Everyday discrimination	8.03	7.38	0–45
*Control variables:*			
Age	45.45	18.09	18–103
Gender			
Male (reference)	48.28%		
Female	51.72%		
Race			
White	80.99%		
Black	9.93%		
Other (reference)	9.08%		
Educational attainment:			
Less than high school	6.14%		
High school or GED	23.37%		
Some college	38.56%		
Bachelor's degree or more	31.93%		
Leukocyte proportion	0.93	0.21	0–1
Assay batch:			
8615	15.77%		
8732	19.17%		
9054	23.09%		
9109	21.30%		
9213	15.04%		
11277	1.48%		
13762	4.15%		
Data collected before the pandemic	39.37%		

^a^ measured in years.

^b^ measured in years of epigenetic aging per chronological years.

^1^ AgeAccelGrim2: M = −0.84, SD = 6.97.

First, we examined the associations between four sources of stress and each of the eight health outcomes independently (unadjusted models). The main results of standardized regression coefficients are presented in Table 2. Our findings show that all sources of stress were significantly associated with accelerated epigenetic aging, and poorer physical and mental health indicators after accounting for basic controls. The size of the associations was substantial. For example, a one standard deviation increase in chronic financial strains was associated with a 0.14 standard deviation increase in accelerated epigenetic aging and a 0.12 standard deviation increase in the pace of epigenetic aging (p < 0.001). To contextualize these effects, a 0.14 standard deviation increase in AgeAccelGrim2 corresponds to approximately one year of accelerated biological aging given a standard deviation of 6.97 for AgeAccelGrim2. Full regression results are presented in S2-S9 Files.

**Table 2:** *Unadjusted* model of psychosocial stressor exposure on epigenetic aging, physical, and mental health outcomes.

	Epigenetic Aging		Physical Health			Mental Health			
	AgeAccel Grim2	Dunedin PACE	Self-Rated Physical Health	Multi morbidity	Pain Interference		Self-Rated Mental Health	Depression Severity	Anxiety Severity
	*B (SE)*	*B (SE)*	*B (SE)*	*B (SE)*	*B (SE)*		*B (SE)*	*B (SE)*	*B (SE)*
ACEs	0.07*** (0.02)	0.06*** (0.02)	0.14*** (0.03)	0.13*** (0.02)	0.13*** (0.03)		0.23*** (0.04)	0.30*** (0.04)	0.27*** (0.04)
Stressful Life Events	0.17*** (0.02)	0.11*** (0.02)	0.15*** (0.03)	0.13*** (0.03)	0.19*** (0.03)		0.17*** (0.04)	0.25*** (0.04)	0.21*** (0.04)
Chronic Financial Strains	0.14*** (0.02)	0.12*** (0.03)	0.21*** (0.03)	0.13*** (0.03)	0.21*** (0.04)		0.30*** (0.04)	0.41*** (0.04)	0.32*** (0.03)
Everyday Discrimination	0.05** (0.02)	0.08** (0.02)	0.19*** (0.02)	0.14*** (0.03)	0.18*** (0.03)		0.25*** (0.03)	0.34*** (0.03)	0.34*** (0.03)

Unadjusted models contain only one source of stress at a time and only adjust for control variables of age, gender, race/ethnicity, education, cell leukocyte proportion, ^a^ assay batch, ^a^ and data collection period

Standardized regression coefficients with standard errors in parentheses

^a^ controlled only in predicting epigenetic aging

* p < 0.05, ** p < 0.01, *** p < 0.001

Next, we examined the adjusted effects of all sources of stress on each of the eight health outcomes by including them simultaneously in the regression models. We find that when all sources of stress were considered simultaneously as predictors of accelerated epigenetic aging, stressful life events showed significant associations with AgeAccelGrim2 (β = 0.14, *P* < 0.001) and PACE (β = 0.06, *P* < 0.05). Similarly, chronic financial strains maintained a significant association with both epigenetic clocks (β = 0.09, *P* < 0.001 and β = 0.09, *P* < 0.01, respectively). ACEs and everyday discrimination, however, were no longer significantly associated with epigenetic aging, when controlling for other stressors.

For physical health outcomes, ACEs were significantly associated with worse self-rated physical health (β = 0.05, *P* < 0.05) and greater multimorbidity (β = 0.06, *P* < 0.05), and stressful life events were only significantly associated with greater pain interference (β = 0.04, *P* < 0.05). Chronic financial strains were a robust predictor across all physical health domains, with significant associations for self-rated physical health (β = 0.16, *P* < 0.001), multimorbidity (β = 0.07, *P* < 0.05), and pain interference (β = 0.15, *P* < 0.001). Similarly, everyday discrimination was significantly associated with worse self-rated physical health (β = 0.13, *P* < 0.001), multimorbidity (β = 0.09, *P* < 0.01), and pain interference (β = 0.12, *P* < 0.001).

For mental health outcomes, ACEs were significantly associated with both depression severity (β = 0.15, *P* < 0.001) and anxiety severity (β = 0.13, *P* < 0.001), as well as poorer self-rated mental health (β = 0.13, *P* < 0.01). Stressful life events showed no significant association with any mental health outcomes. Chronic financial strains showed significant associations with self-rated mental health (β = 0.21, *P* < 0.001), depression severity (β = 0.30, *P* < 0.001), and anxiety severity (β = 0.29, *P* < 0.001). Similarly, everyday discrimination demonstrated significant associations across all mental health domains: self-rated mental health (β = 0.16, *P* < 0.001), depression severity (β = 0.23, *P* < 0.001), and anxiety severity (β = 0.22, *P* < 0.001). Main results are presented in Table 3 below and full regression results with control variables are presented in S10 File.

**Table 3:** *Adjusted* model of psychosocial stressor exposure on epigenetic aging, physical, and mental health outcomes.

	Epigenetic Aging		Physical Health			Mental Health		
	AgeAccel Grim2	Dunedin PACE	Self-Rated Physical Health	Multi morbidity	Pain Interference	Self-Rated Mental Health	Depression Severity	Anxiety Severity
	*B (SE)*	*B (SE)*	*B (SE)*	*B (SE)*	*B (SE)*	*B (SE)*	*B (SE)*	*B (SE)*
ACEs	0.01 (0.02)	0.02 (0.02)	0.05* (0.02)	0.06* (0.03)	0.04 (0.03)	0.13*** (0.04)	0.15*** (0.03)	0.13*** (0.04)
Stressful Life Events	0.14*** (0.02)	0.06* (0.02)	0.03 (0.04)	0.07 (0.05)	0.04* (0.02)	−0.01 (0.04)	0.01 (0.03)	−0.02 (0.04)
Chronic Financial Strain	0.09*** (0.02)	0.09** (0.03)	0.16*** (0.04)	0.07** (0.03)	0.15** (0.03)	0.21*** (0.04)	0.30*** (0.04)	0.29*** (0.05)
Everyday Discrimination	−0.01 (0.02)	0.04 (0.03)	0.13*** (0.03)	0.09** (0.03)	0.12** (0.03)	0.16*** (0.03)	0.23*** (0.03)	0.22*** (0.03)
Model R^2^	0.61	0.42	0.13	0.18	0.11	0.18	0.26	0.25

*Notes*. Adjusted models contain all sources of stress simultaneously and adjust for control variables of age, gender, race/ethnicity, education, cell leukocyte proportion, ^a^ assay batch, ^a^ and data collection period

Standardized regression coefficients with standard errors in parentheses

^a^ Controlled only in predicting epigenetic aging

* p < 0.05, ** p < 0.01, *** p < 0.001

**Fig 1:**
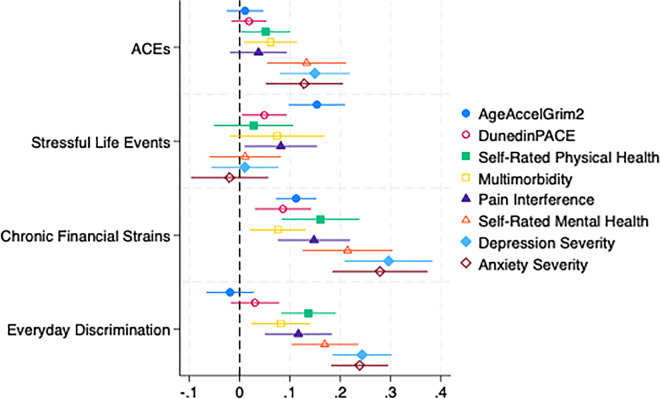
Standardized Regression Coefficients and 95% Confidence Intervals of Psychosocial. Stressor Exposure on Epigenetic Aging, Physical, and Mental Health Outcomes.

To facilitate the comparison of the effects of different stressors on different outcomes, Fig 1 presents standardized regression coefficients with associated 95% confidence intervals. Notably, across a range of health outcomes, ongoing stressors – particularly chronic financial strains and everyday discrimination – emerged as the most consistent predictors of poorer health, especially for mental health outcomes, whereas childhood adversity and stressful life events in adulthood showed smaller impacts once other stressors were considered.

Finally, because about 39% of the survey data were collected during the later stages of the COVID-19 pandemic, we created an indicator of data collection during the COVID-19 period in our analyses to account for the possibility that the pandemic might have enhanced stress exposure and health outcomes. We find that the data collection timing was not associated with accelerated epigenetic aging nor any of the self-reported physical health outcomes. However, we did observe a significant association between the pandemic timing and greater depression and anxiety severity. These results are not surprising; the COVID-19 pandemic represents a unique macro-level psychosocial stressor, and the detrimental mental health effects of specific pandemic-related stressful exposures – including bereavement, economic hardship, loneliness, and relationship strain – as well as their cumulative toll have been well documented in extant scholarship [62–75]. To test the possibility that the pandemic intensified the effect of ongoing financial strains on mental health outcomes in the fully adjusted models, we examined two-way interaction effects and found that the results were not significant. This finding suggests that chronic financial strains affect mental health independently of, and beyond, the acute economic difficulties associated with the pandemic.

## Discussion

Using an age-heterogeneous state-representative sample, the present study provides a comprehensive examination of the impact of four common sources of stress on biological aging, physical health, and mental health in a single analytical framework. When considered independently*,* all four sources of stress – ACEs, stressful life events, chronic financial strains, and everyday discrimination – were significantly associated with accelerated epigenetic aging as well as poorer physical and mental health, supporting established links between stress exposure and health while extending these findings to biological aging processes [4,76].

However, when considered simultaneously, distinct patterns emerged highlighting the relative importance of different sources of stress on health. Specifically, early childhood stressors were no longer significantly associated with epigenetic clocks but remained significantly linked to poorer mental health, corroborating previous research on the impact of ACEs on adulthood self-reported physical and mental health outcomes as well as inflammation when accounting for exposure to adulthood stressors [23,77,78]. Our findings suggest that ACEs may influence biological aging processes indirectly through subsequent life disadvantages in adulthood. In fact, a recently published study has tested this hypothesis empirically and demonstrated that, indeed, greater exposure to stressful life events and ongoing financial difficulties in adulthood accounted for more than 30% of the association between early life stress and epigenetic aging, providing support for stress proliferation across the adult lifespan [79].

Stressful life events were no longer associated with physical and mental health outcomes when other stressors were accounted for, though their effect remained significant for epigenetic aging. The significant association with epigenetic aging is consistent with emerging scholarship [33,80], but previous research has also demonstrated unique significant effects of stressful events on psychological distress [24]. This suggests that stressful events may trigger molecular-level changes that precede observable health impact [4].

Chronic financial strains emerged as the most robust predictor across all health outcomes, but the effects were larger for mental health than physical health outcomes. This pattern indicates that financial difficulties may play a critical role in accelerating biological aging and increasing risk for earlier onset of aging-related disease. In line with the implications of this finding, recent research has demonstrated the slowing of biological aging associated with upward social mobility [81].

Everyday discrimination showed differential effects, remaining significantly associated with mental and physical health outcomes, but not with biological aging, when other stressors were controlled for. This pattern may indicate that ubiquitous discriminatory experiences may not leave a mark at the epigenetic level or produce a weaker signal relative to other sources of stress, as shown in a recent study [42] where financial stress was associated with accelerated epigenetic aging, but discrimination stress was not. We also acknowledge, though, that these stressor categories represent analytically useful distinctions rather than discrete, independent experiences, as life events often catalyze ongoing difficulties and vice versa. In reality, stressors operate within dynamic, interconnected systems of social exposures with important physical and mental health implications.

It is also important to note that the measures for ACEs (range 0 – 10), stressful life events (range 0 – 9), and chronic financial strains (range 0 – 7) are blunter than the everyday discrimination scale (range 0 – 45), which may potentially affect statistical sensitivity, such that the more granular measure may detect smaller variations in exposure. If measurement granularity were driving our findings, we would expect to see the strongest associations between everyday discrimination and our outcomes of interest. However, we found that the measure using the least granular scale – financial strains – is the most robust predictor across all health domains. Moreover, we observed no statistically significant associations between everyday discrimination and epigenetic aging, and the effects of financial strains on depression and anxiety severity were of similar magnitude to those of everyday discrimination. Thus, this increases confidence that our results likely reflect substantive differences across various sources of stress rather than measurement properties of the scales. Nonetheless, the possibility that other indices (e.g., ACEs) were affected by this same issue remain. As such, we encourage future research to examine these associations using measures of comparable granularity.

Several limitations warrant consideration. Due to the cross-sectional nature of the data, we cannot make any causal claims based on our findings. However, the use of epigenetic measures, which reflect the cumulative physiological toll of exposures prior to the onset of morbidity, to an extent addresses the issue of reverse causality and provides some insight into the biological embedding mechanisms of stress over time. Since our sample is limited to Indiana residents, future research could examine these associations with nationally representative data. Further, childhood adversities were measured by asking respondents about experiences before the age of 18 years; therefore, recall bias is a potential concern. We also did not ask when in adulthood the stressors were experienced and how long they lasted, therefore we could not assess the role of recency and precise timing of exposure on health impacts.

Moreover, our study did not examine the full range of important stressor domains, including caregiving burden, occupational stress, relationship strain and loneliness, health-related stress (e.g., managing chronic illness) or neighborhood-level stressors. Each of these has been linked to health in prior research [82–88] and emerging research has documented their importance for accelerated biological aging [89–93]. The absence of these measures means the total effect of stress on health is likely underestimated in our study [27,94]. We should also note that the algorithms for the two DNAm-based measures used in the study – GrimAge2 and DunedinPACE – were originally developed and validated on blood samples, while we applied them to saliva-derived DNA in our analyses. Research [95–97] has demonstrated that biomarkers from both tissues perform similarly, but we encourage scholars to further examine the extent to which tissue type may affect the replicability of findings on the biological embedding of stress in the future. Finally, we would like to acknowledge the potential for selection bias associated with the 30% response rate to the P2P survey. Our response rate aligns with those typical for probability-based household surveys and, as research demonstrates, does not necessarily produce greater bias than surveys achieving higher response rates [98]. As a mitigation strategy, we did use survey weighting procedures in all analyses to adjust for nonresponse bias.

## Conclusion

Our study offers a uniquely comprehensive examination of how varied stress exposures influence health across multiple health domains, spanning from molecular-level epigenetic changes to subjective well-being. Psychosocial stressors were generally more strongly associated with mental health outcomes than physical health outcomes or biological aging, and the effects of stress on biological aging mirrored those on physical health rather than mental health. The robust association between financial strain and all health outcomes considered in the study highlights the need for tailored strategies to mitigate financial insecurity that may increase risk for aging-related complex diseases.

## Supporting information

S1 File Pairwise Correlations of the Main Outcome Variables. * p < 0.05, ** p < 0.01, *** p < 0.001.
(DOCX)

S2 File Standardized Effects from Unadjusted Models of Psychosocial Stressor Exposure on AgeAccelGrim2. Unadjusted models contain only one source of stress at a time and control for covariates. Reference categories are: Male, other, less than high school, batch = 8615, COVID-19 = 0 (data collection before the pandemic). Standardized regression coefficients with standard errors in parentheses. * p < 0.05, ** p < 0.01, *** p < 0.001.
(DOCX)

S3 File Standardized Effects from Unadjusted Models of Psychosocial Stressor Exposure on DunedinPACE. Standardized Effects from Unadjusted Models of Psychosocial Stressor Exposure on AgeAccelGrim2. Notes: Unadjusted models contain only one source of stress at a time and control for covariates. Reference categories are: Male, other, less than high school, batch = 8615, COVID-19 = 0 (data collection before the pandemic). Standardized regression coefficients with standard errors in parentheses. * p < 0.05, ** p < 0.01, *** p < 0.001.
(DOCX)

S4 File Standardized Effects from Unadjusted Models of Psychosocial Stressor Exposure on Self-Rated Health. Unadjusted models contain only one source of stress at a time and control for covariates. Reference categories are: Male, other, less than high school, COVID-19 = 0 (data collection before the pandemic). Standardized regression coefficients with standard errors in parentheses. * p < 0.05, ** p < 0.01, *** p < 0.001.
(DOCX)

S5 File Standardized Effects from Unadjusted Models of Psychosocial Stressor Exposure on Multimorbidity. Unadjusted models contain only one source of stress at a time and control for covariates. Reference categories are: Male, other, less than high school, COVID-19 = 0 (data collection before the pandemic). Standardized regression coefficients with standard errors in parentheses. * p < 0.05, ** p < 0.01, *** p < 0.001.
(DOCX)

S6 File Standardized Effects from Unadjusted Models of Psychosocial Stressor Exposure on Pain Interference. Unadjusted models contain only one source of stress at a time and control for covariates. Reference categories are: Male, other, less than high school, COVID-19 = 0 (data collection before the pandemic). Standardized regression coefficients with standard errors in parentheses. * p < 0.05, ** p < 0.01, *** p < 0.001.
(DOCX)

S7 File Standardized Effects from Unadjusted Models of Psychosocial Stressor Exposure on Self-Rated Mental Health. Unadjusted models contain only one source of stress at a time and control for covariates. Reference categories are: Male, other, less than high school, COVID-19 = 0 (data collection before the pandemic). Standardized regression coefficients with standard errors in parentheses. * p < 0.05, ** p < 0.01, *** p < 0.001.
(DOCX)

S8 File Standardized Effects from Unadjusted Models of Psychosocial Stressor Exposure on Depression Severity. Unadjusted models contain only one source of stress at a time and control for covariates. Reference categories are: Male, other, less than high school, COVID-19 = 0 (data collection before the pandemic). Standardized regression coefficients with standard errors in parentheses. * p < 0.05, ** p < 0.01, *** p < 0.001.
(DOCX)

S9 File Standardized Effects from Unadjusted Models of Psychosocial Stressor Exposure on Anxiety Severity. Unadjusted models contain only one source of stress at a time and control for covariates. Reference categories are: Male, other, less than high school, COVID-19 = 0 (data collection before the pandemic). Standardized regression coefficients with standard errors in parentheses. * p < 0.05, ** p < 0.01, *** p < 0.001.
(DOCX)

S10 File Standardized Effects from Adjusted Models of Psychosocial Stressor Exposure on Epigenetic Aging, Physical, and Mental Health Outcomes. Adjusted models contain all sources of stress simultaneously and adjust for control variables of age, gender, race/ethnicity, education, cell leukocyte proportion, ^a^ assay batch ^a^. Reference categories are: Male, other, less than high school, COVID-19 = 0 (data collection before the pandemic)^. a^Controlled only in predicting epigenetic aging. Standardized regression coefficients with standard errors in parentheses. * p < 0.05, ** p < 0.01, *** p < 0.001.
(DOCX)
