# Supplementary material for: Psychosocial stressors, accelerated biological aging, and multiple morbidities: Evidence from an age-diverse sample
Source: PLoS One. 2026 Mar 6;21(3):e0343987. doi: 10.1371/journal.pone.0343987 (PMC12965587; doi:10.1371/journal.pone.0343987)
Supplement: S1 File — * p < 0.05, ** p < 0.01, *** p < 0.001. (DOCX) [file pone.0343987.s001.docx]

S1 Table. Pairwise Correlations of the Main Outcome Variables

|  | (1) | (2) | (3) | (4) | (5) | (6) | (7) | (8) |
| --- | --- | --- | --- | --- | --- | --- | --- | --- |
| (1) GrimAge2 | 1 |  |  |  |  |  |  |  |
| (2) DunedinPACE | 0.82*** | 1 |  |  |  |  |  |  |
| (3) Physical health | 0.28*** | 0.30*** | 1 |  |  |  |  |  |
| (4) Multimorbidity | 0.21*** | 0.30*** | 0.35*** | 1 |  |  |  |  |
| (5) Pain | 0.24*** | 0.25*** | 0.44*** | 0.36*** | 1 |  |  |  |
| (6) Mental health | 0.20*** | 0.16*** | 0.48*** | 0.13*** | 0.27*** | 1 |  |  |
| (7) Depression severity | 0.19*** | 0.16*** | 0.39*** | 0.14*** | 0.35*** | 0.62*** | 1 |  |
| (8) Anxiety severity | 0.17*** | 0.12*** | 0.34*** | 0.10*** | 0.30*** | 0.56*** | 0.82*** | 1 |

* p<0.05, ** p<0.01, *** p<0.001
