# Supplementary material for: Psychosocial stressors, accelerated biological aging, and multiple morbidities: Evidence from an age-diverse sample
Source: PLoS One. 2026 Mar 6;21(3):e0343987. doi: 10.1371/journal.pone.0343987 (PMC12965587; doi:10.1371/journal.pone.0343987)
Supplement: S2 File — Unadjusted models contain only one source of stress at a time and control for covariates. Reference categories are: Male, other, less than high school, batch = 8615, COVID-19 = 0 (data collection before the pandemic). Standardized regression coefficients with standard errors in parentheses. * p < 0.05, ** p < 0.01, *** p < 0.001. (DOCX) [file pone.0343987.s002.docx]

S2 Table. Standardized Effects from Unadjusted Models of Psychosocial Stressor Exposure on AgeAccelGrim2

|  | *B (SE)* | *B (SE)* | *B (SE)* | *B (SE)* |
| --- | --- | --- | --- | --- |
| ACEs | 0.074*** |  |  |  |
|  | (0.016) |  |  |  |
| Stressful Life Events |  | 0.169*** |  |  |
|  |  | (0.021) |  |  |
| Chronic Financial Strains |  |  | 0.136*** |  |
|  |  |  | (0.021) |  |
| Everyday Discrimination |  |  |  | 0.054** |
|  |  |  |  | (0.018) |
| Age | -0.006*** | -0.011*** | -0.005*** | -0.006*** |
|  | (0.001) | (0.001) | (0.001) | (0.001) |
| Female | -0.010 | 0.032 | -0.007 | 0.015 |
|  | (0.037) | (0.035) | (0.035) | (0.035) |
| White | -0.106 | -0.109 | -0.093 | -0.115 |
|  | (0.064) | (0.068) | (0.062) | (0.063) |
| Black | 0.199* | 0.117 | 0.163 | 0.179* |
|  | (0.088) | (0.093) | (0.088) | (0.086) |
| High school or GED | -0.059 | -0.021 | -0.035 | -0.052 |
|  | (0.071) | (0.066) | (0.071) | (0.070) |
| Some college or Associate’s | -0.242** | -0.176* | -0.195* | -0.240** |
|  | (0.084) | (0.075) | (0.080) | (0.082) |
| College or more | -0.418*** | -0.334*** | -0.361*** | -0.444*** |
|  | (0.076) | (0.068) | (0.072) | (0.074) |
| Batch=8732 | -0.053 | -0.052 | -0.056 | -0.053 |
|  | (0.060) | (0.062) | (0.062) | (0.061) |
| Batch=9054 | -0.041 | -0.039 | -0.040 | -0.047 |
|  | (0.047) | (0.049) | (0.047) | (0.046) |
| Batch=9109 | -0.014 | -0.023 | -0.023 | -0.012 |
|  | (0.064) | (0.062) | (0.061) | (0.063) |
| Batch=9213 | -0.109* | -0.110* | -0.124* | -0.112* |
|  | (0.052) | (0.050) | (0.050) | (0.051) |
| Batch=11277 | -0.089 | -0.063 | -0.066 | -0.092 |
|  | (0.098) | (0.092) | (0.096) | (0.101) |
| Batch=13762 | -0.138 | -0.128 | -0.126 | -0.145 |
|  | (0.133) | (0.133) | (0.138) | (0.129) |
| Leukocyte Proportion | -0.764*** | -0.754*** | -0.753*** | -0.769*** |
|  | (0.018) | (0.018) | (0.018) | (0.018) |
| COVID-19 (1 = Yes) | 0.019 | 0.025 | 0.022 | 0.017 |
|  | (0.029) | (0.027) | (0.028) | (0.027) |
| R-squared | 0.585 | 0.603 | 0.596 | 0.582 |

Notes: Unadjusted models contain only one source of stress at a time and control for covariates

Reference categories are: Male, other, less than high school, Batch = 8615, COVID-19 = 0 (data collection before the pandemic)

Standardized regression coefficients with standard errors in parentheses

* p<0.05, ** p<0.01, *** p<0.001
