# Supplementary material for: Psychosocial stressors, accelerated biological aging, and multiple morbidities: Evidence from an age-diverse sample
Source: PLoS One. 2026 Mar 6;21(3):e0343987. doi: 10.1371/journal.pone.0343987 (PMC12965587; doi:10.1371/journal.pone.0343987)
Supplement: S3 File — Standardized Effects from Unadjusted Models of Psychosocial Stressor Exposure on AgeAccelGrim2. Notes: Unadjusted models contain only one source of stress at a time and control for covariates. Reference categories are: Male, other, less than high school, batch = 8615, COVID-19 = 0 (data collection before the pandemic). Standardized regression coefficients with standard errors in parentheses. * p < 0.05, ** p < 0.01, *** p < 0.001. (DOCX) [file pone.0343987.s003.docx]

S3 Table. Standardized Effects from Unadjusted Models of Psychosocial Stressor Exposure on DunedinPACE

|  | *B (SE)* | *B (SE)* | *B (SE)* | *B (SE)* |
| --- | --- | --- | --- | --- |
| ACEs | 0.068*** |  |  |  |
|  | (0.017) |  |  |  |
| Stressful Life Events |  | 0.109*** |  |  |
|  |  | (0.024) |  |  |
| Chronic Financial Strains |  |  | 0.122*** |  |
|  |  |  | (0.026) |  |
| Everyday Discrimination |  |  |  | 0.081** |
|  |  |  |  | (0.026) |
| Age | 0.004** | 0.001 | 0.006*** | 0.005** |
|  | (0.002) | (0.002) | (0.001) | (0.002) |
| Female | 0.153*** | 0.186*** | 0.157*** | 0.178*** |
|  | (0.040) | (0.039) | (0.040) | (0.039) |
| White | -0.211** | -0.219** | -0.200** | -0.215** |
|  | (0.073) | (0.076) | (0.074) | (0.072) |
| Black | 0.224* | 0.167 | 0.191* | 0.202* |
|  | (0.095) | (0.099) | (0.095) | (0.091) |
| High school or GED | -0.015 | 0.012 | 0.007 | -0.009 |
|  | (0.106) | (0.110) | (0.110) | (0.105) |
| Some college or Associate's | -0.159 | -0.120 | -0.125 | -0.160 |
|  | (0.110) | (0.108) | (0.111) | (0.107) |
| College or more | -0.352** | -0.304** | -0.302** | -0.379*** |
|  | (0.106) | (0.100) | (0.106) | (0.103) |
| Batch=8732 | -0.127** | -0.127** | -0.130** | -0.124** |
|  | (0.046) | (0.045) | (0.046) | (0.045) |
| Batch=9054 | -0.209*** | -0.209*** | -0.209*** | -0.216*** |
|  | (0.054) | (0.055) | (0.052) | (0.050) |
| Batch=9109 | -0.078 | -0.083 | -0.087 | -0.077 |
|  | (0.053) | (0.054) | (0.050) | (0.051) |
| Batch=9213 | -0.311*** | -0.311*** | -0.324*** | -0.316*** |
|  | (0.075) | (0.079) | (0.074) | (0.074) |
| Batch=11277 | 0.170 | 0.180 | 0.190 | 0.178 |
|  | (0.192) | (0.191) | (0.192) | (0.195) |
| Batch=13762 | -0.260 | -0.256 | -0.249 | -0.265* |
|  | (0.135) | (0.138) | (0.138) | (0.130) |
| Leukocyte Proportion | -0.603*** | -0.598*** | -0.594*** | -0.608*** |
|  | (0.044) | (0.047) | (0.046) | (0.046) |
| COVID-19 (1 = Yes) | -0.011 | -0.009 | -0.008 | -0.012 |
|  | (0.041) | (0.038) | (0.039) | (0.037) |
| R-squared | 0.404 | 0.409 | 0.412 | 0.406 |

Notes: Unadjusted models contain only one source of stress at a time and control for covariates

Reference categories are: Male, other, less than high school, Batch = 8615, COVID-19 = 0 (data collection before the pandemic)

Standardized regression coefficients with standard errors in parentheses

* p<0.05, ** p<0.01, *** p<0.001
