# Supplementary material for: Psychosocial stressors, accelerated biological aging, and multiple morbidities: Evidence from an age-diverse sample
Source: PLoS One. 2026 Mar 6;21(3):e0343987. doi: 10.1371/journal.pone.0343987 (PMC12965587; doi:10.1371/journal.pone.0343987)
Supplement: S4 File — Unadjusted models contain only one source of stress at a time and control for covariates. Reference categories are: Male, other, less than high school, COVID-19 = 0 (data collection before the pandemic). Standardized regression coefficients with standard errors in parentheses. * p < 0.05, ** p < 0.01, *** p < 0.001. (DOCX) [file pone.0343987.s004.docx]

S4 Table. Standardized Effects from Unadjusted Models of Psychosocial Stressor Exposure on Self-Rated Health

|  | *B (SE)* | *B (SE)* | *B (SE)* | *B (SE)* |
| --- | --- | --- | --- | --- |
| ACEs | 0.136*** |  |  |  |
|  | (0.024) |  |  |  |
| Stressful Life Events |  | 0.148*** |  |  |
|  |  | (0.035) |  |  |
| Chronic Financial Strains |  |  | 0.214*** |  |
|  |  |  | (0.033) |  |
| Everyday Discrimination |  |  |  | 0.190*** |
|  |  |  |  | (0.022) |
| Age | 0.003* | -0.001 | 0.006*** | 0.005*** |
|  | (0.001) | (0.002) | (0.002) | (0.001) |
| Female | 0.180*** | 0.237*** | 0.191*** | 0.232*** |
|  | (0.051) | (0.047) | (0.048) | (0.051) |
| White | -0.117 | -0.139 | -0.105 | -0.118 |
|  | (0.129) | (0.134) | (0.129) | (0.126) |
| Black | -0.032 | -0.119 | -0.096 | -0.079 |
|  | (0.144) | (0.147) | (0.148) | (0.140) |
| High school or GED | -0.219 | -0.176 | -0.173 | -0.211 |
|  | (0.112) | (0.123) | (0.124) | (0.122) |
| Some college or Associate's | -0.438*** | -0.383** | -0.373** | -0.443*** |
|  | (0.102) | (0.113) | (0.109) | (0.111) |
| College or more | -0.680*** | -0.625*** | -0.587*** | -0.741*** |
|  | (0.116) | (0.131) | (0.125) | (0.124) |
| COVID-19 (1 = Yes) | -0.018 | -0.016 | -0.012 | -0.019 |
|  | (0.059) | (0.054) | (0.058) | (0.054) |
| R-squared | 0.089 | 0.088 | 0.109 | 0.106 |

Notes: Unadjusted models contain only one source of stress at a time and control for covariates

Reference categories are: Male, other, less than high school, COVID-19 = 0 (data collection before the pandemic)

Standardized regression coefficients with standard errors in parentheses

* p<0.05, ** p<0.01, *** p<0.001
