# Supplementary material for: Psychosocial stressors, accelerated biological aging, and multiple morbidities: Evidence from an age-diverse sample
Source: PLoS One. 2026 Mar 6;21(3):e0343987. doi: 10.1371/journal.pone.0343987 (PMC12965587; doi:10.1371/journal.pone.0343987)
Supplement: S5 File — Unadjusted models contain only one source of stress at a time and control for covariates. Reference categories are: Male, other, less than high school, COVID-19 = 0 (data collection before the pandemic). Standardized regression coefficients with standard errors in parentheses. * p < 0.05, ** p < 0.01, *** p < 0.001. (DOCX) [file pone.0343987.s005.docx]

S5 Table. Standardized Effects from Unadjusted Models of Psychosocial Stressor Exposure on Multimorbidity

|  | *B (SE)* | *B (SE)* | *B (SE)* | *B (SE)* |
| --- | --- | --- | --- | --- |
| ACEs | 0.127*** |  |  |  |
|  | (0.023) |  |  |  |
| Stressful Life Events |  | 0.147*** |  |  |
|  |  | (0.040) |  |  |
| Chronic Financial Strains |  |  | 0.134*** |  |
|  |  |  | (0.025) |  |
| Everyday Discrimination |  |  |  | 0.142*** |
|  |  |  |  | (0.027) |
| Age | 0.020*** | 0.015*** | 0.021*** | 0.021*** |
|  | (0.001) | (0.001) | (0.001) | (0.001) |
| Female | 0.016 | 0.070 | 0.034 | 0.061 |
|  | (0.046) | (0.050) | (0.053) | (0.049) |
| White | -0.011 | -0.031 | -0.014 | -0.018 |
|  | (0.087) | (0.088) | (0.086) | (0.085) |
| Black | 0.000 | -0.084 | -0.048 | -0.039 |
|  | (0.107) | (0.109) | (0.107) | (0.098) |
| High school or GED | -0.154 | -0.111 | -0.121 | -0.145 |
|  | (0.126) | (0.121) | (0.125) | (0.125) |
| Some college or Associate's | -0.229 | -0.174 | -0.188 | -0.233 |
|  | (0.147) | (0.137) | (0.143) | (0.141) |
| College or more | -0.237 | -0.179 | -0.192 | -0.290* |
|  | (0.137) | (0.129) | (0.138) | (0.131) |
| COVID-19 (1 = Yes) | 0.059 | 0.061 | 0.060 | 0.057 |
|  | (0.049) | (0.050) | (0.050) | (0.052) |
| R-squared | 0.161 | 0.162 | 0.161 | 0.166 |

Notes: Unadjusted models contain only one source of stress at a time and control for covariates

Reference categories are: Male, other, less than high school, COVID-19 = 0 (data collection before the pandemic)

Standardized regression coefficients with standard errors in parentheses

* p<0.05, ** p<0.01, *** p<0.001
