# Supplementary material for: Psychosocial stressors, accelerated biological aging, and multiple morbidities: Evidence from an age-diverse sample
Source: PLoS One. 2026 Mar 6;21(3):e0343987. doi: 10.1371/journal.pone.0343987 (PMC12965587; doi:10.1371/journal.pone.0343987)
Supplement: S6 File — Unadjusted models contain only one source of stress at a time and control for covariates. Reference categories are: Male, other, less than high school, COVID-19 = 0 (data collection before the pandemic). Standardized regression coefficients with standard errors in parentheses. * p < 0.05, ** p < 0.01, *** p < 0.001. (DOCX) [file pone.0343987.s006.docx]

S6 Table. Standardized Effects from Unadjusted Models of Psychosocial Stressor Exposure on Pain Interference

|  | *B (SE)* | *B (SE)* | *B (SE)* | *B (SE)* |
| --- | --- | --- | --- | --- |
| ACEs | 0.132*** |  |  |  |
|  | (0.031) |  |  |  |
| Stressful Life Events |  | 0.186*** |  |  |
|  |  | (0.027) |  |  |
| Chronic Financial Strains |  |  | 0.214*** |  |
|  |  |  | (0.035) |  |
| Everyday Discrimination |  |  |  | 0.182*** |
|  |  |  |  | (0.028) |
| Age | 0.007*** | 0.001 | 0.009*** | 0.008*** |
|  | (0.001) | (0.002) | (0.002) | (0.002) |
| Female | 0.034 | 0.095 | 0.044 | 0.084 |
|  | (0.053) | (0.048) | (0.047) | (0.046) |
| White | 0.091 | 0.073 | 0.104 | 0.090 |
|  | (0.091) | (0.083) | (0.082) | (0.082) |
| Black | 0.135 | 0.034 | 0.072 | 0.090 |
|  | (0.114) | (0.112) | (0.104) | (0.109) |
| High school or GED | -0.359* | -0.308* | -0.313* | -0.351* |
|  | (0.143) | (0.141) | (0.141) | (0.145) |
| Some college or Associate's | -0.371* | -0.302* | -0.306* | -0.376* |
|  | (0.148) | (0.141) | (0.142) | (0.149) |
| College or more | -0.608*** | -0.526*** | -0.513*** | -0.666*** |
|  | (0.140) | (0.136) | (0.143) | (0.136) |
| COVID-19 (1 = Yes) | -0.043 | -0.038 | -0.037 | -0.044 |
|  | (0.075) | (0.068) | (0.074) | (0.071) |
| R-squared | 0.066 | 0.076 | 0.089 | 0.082 |

Notes: Unadjusted models contain only one source of stress at a time and control for covariates

Reference categories are: Male, other, less than high school, COVID-19 = 0 (data collection before the pandemic)

Standardized regression coefficients with standard errors in parentheses

* p<0.05, ** p<0.01, *** p<0.001
