# Supplementary material for: Psychosocial stressors, accelerated biological aging, and multiple morbidities: Evidence from an age-diverse sample
Source: PLoS One. 2026 Mar 6;21(3):e0343987. doi: 10.1371/journal.pone.0343987 (PMC12965587; doi:10.1371/journal.pone.0343987)
Supplement: S7 File — Unadjusted models contain only one source of stress at a time and control for covariates. Reference categories are: Male, other, less than high school, COVID-19 = 0 (data collection before the pandemic). Standardized regression coefficients with standard errors in parentheses. * p < 0.05, ** p < 0.01, *** p < 0.001. (DOCX) [file pone.0343987.s007.docx]

S7 Table. Standardized Effects from Unadjusted Models of Psychosocial Stressor Exposure on Self-Rated Mental Health

|  | *B (SE)* | *B (SE)* | *B (SE)* | *B (SE)* |
| --- | --- | --- | --- | --- |
| ACEs | 0.234*** |  |  |  |
|  | (0.036) |  |  |  |
| Stressful Life Events |  | 0.169*** |  |  |
|  |  | (0.035) |  |  |
| Chronic Financial Strains |  |  | 0.296*** |  |
|  |  |  | (0.039) |  |
| Everyday Discrimination |  |  |  | 0.182*** |
|  |  |  |  | (0.028) |
| Age | -0.006*** | -0.012*** | -0.003 | 0.008*** |
|  | (0.002) | (0.002) | (0.002) | (0.002) |
| Female | 0.162** | 0.249*** | 0.190*** | 0.084 |
|  | (0.057) | (0.049) | (0.048) | (0.046) |
| White | 0.067 | 0.023 | 0.073 | 0.090 |
|  | (0.086) | (0.102) | (0.103) | (0.082) |
| Black | -0.020 | -0.133 | -0.116 | 0.090 |
|  | (0.140) | (0.156) | (0.151) | (0.109) |
| High school or GED | -0.107 | -0.050 | -0.039 | -0.351* |
|  | (0.149) | (0.159) | (0.145) | (0.145) |
| Some college or Associate's | -0.355** | -0.293* | -0.266* | -0.376* |
|  | (0.115) | (0.121) | (0.111) | (0.149) |
| College or more | -0.490*** | -0.450** | -0.375** | -0.666*** |
|  | (0.117) | (0.132) | (0.126) | (0.136) |
| COVID-19 (1 = Yes) | 0.086 | 0.083 | 0.091 | -0.044 |
|  | (0.058) | (0.051) | (0.054) | (0.071) |
| R-squared | 0.119 | 0.090 | 0.138 | 0.082 |

Notes: Unadjusted models contain only one source of stress at a time and control for covariates

Reference categories are: Male, other, less than high school, COVID-19 = 0 (data collection before the pandemic)

Standardized regression coefficients with standard errors in parentheses

* p<0.05, ** p<0.01, *** p<0.001
