# Supplementary material for: Psychosocial stressors, accelerated biological aging, and multiple morbidities: Evidence from an age-diverse sample
Source: PLoS One. 2026 Mar 6;21(3):e0343987. doi: 10.1371/journal.pone.0343987 (PMC12965587; doi:10.1371/journal.pone.0343987)
Supplement: S8 File — Unadjusted models contain only one source of stress at a time and control for covariates. Reference categories are: Male, other, less than high school, COVID-19 = 0 (data collection before the pandemic). Standardized regression coefficients with standard errors in parentheses. * p < 0.05, ** p < 0.01, *** p < 0.001. (DOCX) [file pone.0343987.s008.docx]

S8 Table. Standardized Effects from Unadjusted Models of Psychosocial Stressor Exposure on Depression Severity

|  | *B (SE)* | *B (SE)* | *B (SE)* | *B (SE)* |
| --- | --- | --- | --- | --- |
| ACEs | 0.303*** |  |  |  |
|  | (0.035) |  |  |  |
| Stressful Life Events |  | 0.250*** |  |  |
|  |  | (0.035) |  |  |
| Chronic Financial Strains |  |  | 0.413*** |  |
|  |  |  | (0.040) |  |
| Everyday Discrimination |  |  |  | 0.344*** |
|  |  |  |  | (0.030) |
| Age | -0.006*** | -0.015*** | -0.002 | -0.003* |
|  | (0.002) | (0.002) | (0.002) | (0.002) |
| Female | 0.139* | 0.256*** | 0.171*** | 0.248*** |
|  | (0.057) | (0.050) | (0.046) | (0.051) |
| White | 0.174 | 0.119 | 0.188 | 0.159 |
|  | (0.102) | (0.098) | (0.105) | (0.099) |
| Black | -0.038 | -0.198 | -0.169 | -0.133 |
|  | (0.131) | (0.128) | (0.129) | (0.121) |
| High school or GED | -0.144 | -0.064 | -0.052 | -0.124 |
|  | (0.154) | (0.160) | (0.155) | (0.156) |
| Some college or Associate's | -0.128 | -0.036 | -0.003 | -0.138 |
|  | (0.126) | (0.121) | (0.118) | (0.120) |
| College or more | -0.193 | -0.122 | -0.026 | -0.321* |
|  | (0.138) | (0.144) | (0.140) | (0.136) |
| COVID-19 (1 = Yes) | 0.086 | 0.084 | 0.095 | 0.080 |
|  | (0.059) | (0.050) | (0.054) | (0.052) |
| R-squared | 0.130 | 0.092 | 0.183 | 0.156 |

Notes: Unadjusted models contain only one source of stress at a time and control for covariates

Reference categories are: Male, non-White, less than high school, COVID-19 = 0 (data collection before the pandemic)

Standardized regression coefficients with standard errors in parentheses

* p<0.05, ** p<0.01, *** p<0.001
