# Supplementary material for: Psychosocial stressors, accelerated biological aging, and multiple morbidities: Evidence from an age-diverse sample
Source: PLoS One. 2026 Mar 6;21(3):e0343987. doi: 10.1371/journal.pone.0343987 (PMC12965587; doi:10.1371/journal.pone.0343987)
Supplement: S9 File — Unadjusted models contain only one source of stress at a time and control for covariates. Reference categories are: Male, other, less than high school, COVID-19 = 0 (data collection before the pandemic). Standardized regression coefficients with standard errors in parentheses. * p < 0.05, ** p < 0.01, *** p < 0.001. (DOCX) [file pone.0343987.s009.docx]

S9 Table. Standardized Effects from Unadjusted Models of Psychosocial Stressor Exposure on Anxiety Severity

|  | *B (SE)* | *B (SE)* | *B (SE)* | *B (SE)* |
| --- | --- | --- | --- | --- |
| ACEs | 0.268*** |  |  |  |
|  | (0.037) |  |  |  |
| Stressful Life Events |  | 0.206*** |  |  |
|  |  | (0.037) |  |  |
| Chronic Financial Strains |  |  | 0.382*** |  |
|  |  |  | (0.042) |  |
| Everyday Discrimination |  |  |  | 0.319*** |
|  |  |  |  | (0.030) |
| Age | -0.009*** | -0.016*** | -0.004* | -0.006*** |
|  | (0.002) | (0.002) | (0.002) | (0.002) |
| Female | 0.167** | 0.268*** | 0.193*** | 0.265*** |
|  | (0.054) | (0.057) | (0.052) | (0.054) |
| White | 0.027 | -0.023 | 0.043 | 0.016 |
|  | (0.093) | (0.092) | (0.089) | (0.083) |
| Black | -0.079 | -0.214 | -0.198 | -0.164 |
|  | (0.113) | (0.116) | (0.105) | (0.102) |
| High school or GED | -0.072 | -0.004 | 0.012 | -0.054 |
|  | (0.162) | (0.168) | (0.157) | (0.168) |
| Some college or Associate's | -0.112 | -0.036 | 0.004 | -0.121 |
|  | (0.107) | (0.105) | (0.100) | (0.110) |
| College or more | -0.138 | -0.086 | 0.020 | -0.253 |
|  | (0.128) | (0.139) | (0.132) | (0.130) |
| COVID-19 (1 = Yes) | 0.142* | 0.139* | 0.151* | 0.137** |
|  | (0.065) | (0.059) | (0.060) | (0.051) |
| R-squared | 0.132 | 0.097 | 0.183 | 0.161 |

Notes: Unadjusted models contain only one source of stress at a time and control for covariates

Reference categories are: Male, other, less than high school, COVID-19 = 0 (data collection before the pandemic)

Standardized regression coefficients with standard errors in parentheses

* p<0.05, ** p<0.01, *** p<0.001
