# Supplementary material for: Psychosocial stressors, accelerated biological aging, and multiple morbidities: Evidence from an age-diverse sample
Source: PLoS One. 2026 Mar 6;21(3):e0343987. doi: 10.1371/journal.pone.0343987 (PMC12965587; doi:10.1371/journal.pone.0343987)
Supplement: S10 File — Adjusted models contain all sources of stress simultaneously and adjust for control variables of age, gender, race/ethnicity, education, cell leukocyte proportion, a assay batch a. Reference categories are: Male, other, less than high school, COVID-19 = 0 (data collection before the pandemic). aControlled only in predicting epigenetic aging. Standardized regression coefficients with standard errors in parentheses. * p < 0.05, ** p < 0.01, *** p < 0.001. (DOCX) [file pone.0343987.s010.docx]

S10 Table. Standardized Effects from Adjusted Models of Psychosocial Stressor Exposure on Epigenetic Aging, Physical, and Mental Health Outcomes

|  | AgeAccel  Grim2 | PACE | Physical  Health | Multimorb  idity | Pain | Mental  Health | Dep. Severity | Anx.  Severity |
| --- | --- | --- | --- | --- | --- | --- | --- | --- |
| ACEs | 0.014 | 0.017 | 0.048* | 0.063* | 0.036 | 0.131*** | 0.153*** | 0.131*** |
|  | (0.016) | (0.018) | (0.024) | (0.027) | (0.029) | (0.036) | (0.033) | (0.036) |
| Stressful Life Events | 0.138*** | 0.060* | 0.030 | 0.068 |  | -0.008 | 0.011 | -0.019 |
|  | (0.021) | (0.025) | (0.038) | (0.048) |  | (0.037) | (0.032) | (0.037) |
| Chronic Financial Strains | 0.094*** | 0.089** | 0.156*** | 0.071* | 0.149*** | 0.215*** | 0.302*** | 0.289*** |
|  | (0.021) | (0.028) | (0.039) | (0.027) | (0.034) | (0.043) | (0.044) | (0.048) |
| Everyday Discrimination | -0.009 | 0.040 | 0.134*** | 0.089** | 0.116*** | 0.165*** | 0.232*** | 0.224*** |
|  | (0.018) | (0.026) | (0.025) | (0.028) | (0.032) | (0.032) | (0.027) | (0.028) |
| Age | -0.009*** | 0.005** | 0.007*** | 0.020*** | 0.008*** | -0.000 | 0.001 | -0.001 |
|  | (0.001) | (0.002) | (0.002) | (0.002) | (0.002) | (0.002) | (0.002) | (0.002) |
| Female |  | 0.167*** | 0.199*** | 0.040 | 0.061 | 0.175** | 0.161** | 0.183*** |
|  | (0.034) |  |  |  |  |  |  |  |
| White | -0.088 | -0.191* | -0.081 | 0.009 | 0.125 | 0.115 | 0.242* | 0.092 |
|  | (0.067) | (0.076) | (0.125) | (0.088) | (0.081) | (0.088) | (0.101) | (0.082) |
| Black | 0.119 | 0.173 | -0.104 | -0.062 | 0.044 | -0.092 | -0.151 | -0.173 |
|  | (0.094) | (0.098) | (0.145) | (0.109) | (0.107) | (0.132) | (0.114) | (0.094) |
| High school or GED | -0.017 | 0.011 | -0.184 | -0.125 | -0.313* | -0.070 | -0.087 | -0.024 |
|  | (0.067) | (0.110) | (0.122) | (0.122) | (0.131) | (0.140) | (0.136) | (0.148) |
| Some college or Associate’s | -0.155* | -0.140 | -0.352** | -0.198 | -0.313* | -0.281* | -0.023 | -0.014 |
|  | (0.072) | (0.101) | (0.120) | (0.143) | (0.139) | (0.112) | (0.108) | (0.096) |
| College or more | -0.295*** | -0.283* | -0.602*** | -0.175 | -0.501*** | -0.407*** | -0.063 | -0.032 |
|  | (0.066) | (0.107) | (0.134) | (0.132) | (0.134) | (0.115) | (0.117) | (0.122) |
| Batch=8732 | -0.052 | -0.124** |  |  |  |  |  |  |
|  | (0.062) | (0.046) |  |  |  |  |  |  |
| Batch=9054 | -0.035 | -0.208*** |  |  |  |  |  |  |
|  | (0.049) | (0.052) |  |  |  |  |  |  |
| Batch=9109 | -0.031 | -0.091 |  |  |  |  |  |  |
|  | (0.061) | (0.051) |  |  |  |  |  |  |
| Batch=9213 | -0.120* | -0.324*** |  |  |  |  |  |  |
|  | (0.049) | (0.075) |  |  |  |  |  |  |
| Batch=11277 | -0.040 | 0.215 |  |  |  |  |  |  |
|  | (0.091) | (0.198) |  |  |  |  |  |  |
| Batch=13762 | -0.116 | -0.244 |  |  |  |  |  |  |
|  | (0.135) | (0.131) |  |  |  |  |  |  |
| Leukocyte Proportion | -0.745*** | -0.591*** |  |  |  |  |  |  |
|  | (0.018) | (0.046) |  |  |  |  |  |  |
| COVID-19 (1 = Yes) | 0.029 | -0.004 | -0.005 | 0.068 | -0.028 | 0.100 | 0.107* | 0.161** |
|  | (0.027) | (0.038) | (0.054) | (0.050) | (0.071) | (0.053) | (0.053) | (0.055) |
| R-squared | 0.611 | 0.418 | 0.131 | 0.183 | 0.114 | 0.182 | 0.263 | 0.250 |

*Notes*. Adjusted models contain all sources of stress simultaneously and adjust for control variables of age, gender, race/ethnicity, education, cell leukocyte proportion, ^a^ assay batch ^a^

Reference categories are: Male, other, less than high school, COVID-19 = 0 (data collection before the pandemic)

^a^Controlled only in predicting epigenetic aging

Standardized regression coefficients with standard errors in parentheses

* p<0.05, ** p<0.01, *** p<0.001
